# TAK-242 (Resatorvid) inhibits proinflammatory cytokine production through the inhibition of NF-κB signaling pathway in fibroblast-like synoviocytes in osteoarthritis patients

**DOI:** 10.1186/s42358-024-00385-9

**Published:** 2024-06-07

**Authors:** Mohammadreza Khomeijani-Farahani, Jafar karami, Elham Farhadi, Samaneh Soltani, Ali-Akbar Delbandi, Mehdi Shekarabi, Mohammad Naghi Tahmasebi, Arash Sharafat Vaziri, Ahmadreza Jamshidi, Mahdi Mahmoudi, Masoomeh Akhlaghi

**Affiliations:** 1 Rheumatology Research Center, Tehran University of Medical Sciences, Tehran, Iran; 2 Students Scientific Research Center, Tehran University of Medical Sciences, Tehran, Iran; 3 Molecular and Medicine Research Center, Khomein University of Medical Sciences, Khomein, Iran; 4 Research Center for Chronic Inflammatory Diseases, Tehran University of Medical Sciences, Tehran, Iran; 5 Department of Immunology, School of Medicine, Iran University of Medical Sciences, Tehran, Iran; 6 Immunology Research Center, Institute of Immunology and Infectious Diseases, Iran University of Medical Sciences, Tehran, Iran; 7 Department of Orthopedics, Division of Knee Surgery, Shariati Hospital, Tehran University of Medical Sciences, Tehran, Iran; 8 Rheumatology Research Center, Shariati Hospital, Kargar Ave., Tehran, Iran

**Keywords:** Fibroblast-like synoviocytes, IL1-β, IL-6, Osteoarthritis, TAK-242, TLR4, TNF-*α*

## Abstract

**Background:**

Fibroblast-like synoviocytes (FLSs) are involved in osteoarthritis (OA) pathogenesis through pro-inflammatory cytokine production. TAK-242, a TLR4 blocker, has been found to have a significant impact on the gene expression profile of pro-inflammatory cytokines such as IL1-β, IL-6, TNF-α, and TLR4, as well as the phosphorylation of Ikβα, a regulator of the NF-κB signaling pathway, in OA-FLSs. This study aims to investigate this effect because TLR4 plays a crucial role in inflammatory responses.

**Materials and methods:**

Ten OA patients’ synovial tissues were acquired, and isolated FLSs were cultured in DMEM in order to assess the effectiveness of TAK-242. The treated FLSs with TAK-242 and Lipopolysaccharides (LPS) were analyzed for the mRNA expression level of *IL1-β, IL-6, TNF-α*, and *TLR4* levels by Real-Time PCR. Besides, we used western blot to assess the protein levels of Ikβα and pIkβα.

**Results:**

The results represented that TAK-242 effectively suppressed the gene expression of inflammatory cytokines *IL1-β*, *IL-6*, *TNF-α*, and *TLR4* which were overexpressed upon LPS treatment. Additionally, TAK-242 inhibited the phosphorylation of Ikβα which was increased by LPS treatment.

**Conclusion:**

According to our results, TAK-242 shows promising inhibitory effects on TLR4-mediated inflammatory responses in OA-FLSs by targeting the NF-κB pathway. TLR4 inhibitors, such as TAK-242, may be useful therapeutic agents to reduce inflammation and its associated complications in OA patients, since traditional and biological treatments may not be adequate for all of them.

## Introduction

Osteoarthritis (OA) is a progressive chronic and frequent kind of arthritis, affecting about 15% of the population, and there is no disease-modifying treatment available [1]. OA progression leads to synovial membrane changes, including fibrogenesis [2]. Also, resident synoviocytes are thought to have a prominent role in the pathogenesis of OA, and considering their fibroblast lineage, fibroblast-like synoviocytes (FLSs) are contemplated as critical facilitators of synovial fibrosis [3]. Located primarily in the intimal layer of the synovium, FLSs are the predominant cell type and constitute an intimal layer by interacting with the extracellular matrix (ECM) and other synovialcomponents [4].

Inflammation significantly impacts osteoarthritis development. Following inflammation, the release of major inflammatory signals such as interleukin-1 beta (IL-1β) is stimulated, which consequently leads to the activation and promotion of signaling pathways, such as nuclear factor kappa-light-chain-enhancer of activated B cells (NF-κB), phosphoinositide 3-kinase/protein kinase B (PI3K/AKT), and mitogen-activated protein kinase (MAPK) in a broad number of immune cell types. Following a complex sequence of events, further inflammatory molecules are produced, and eventually, collagenase-like matrix metalloproteinase-13 (MMP-13) deteriorates the extracellular matrix [5, 6]. The NF-κB transcription factor is the master regulator of the inflammatory response and is essential for the homeostasis of the immune system. In mammals, the NF-κB/Rel family comprises five members: p50, p52, p65 (Rel-A), c-Rel, and Rel-B proteins [7]. Phosphorylation plays a critical role in the activation of NF-κB downstream of all these stimuli. The inhibitor of NF-κB alpha (IκBα) protein is an important regulator of the transcription factor NF-κB. NF-κB is trapped by its bonds to IκB molecules in the cytoplasm, and when the IκB is phosphorylated and degraded, the subunits of NF-κB, which are p50 and p65, are allowed to enter the nucleus and activate target genes [8].

Dysregulation of the endo-lysosomal compartment contributes to the development of diverse human diseases [9]. CD14, a cell surface differentiation marker, is present on monocytes, macrophages, dendritic cells (DCs), and neutrophils, where it acts as a receptor for lipopolysaccharide (LPS). Because CD14 lacks a transmembrane domain, it is unable to initiate signaling responses by itself. LPS bound to CD14 is transferred to the Toll-like receptor-4 (TLR-4)-myeloid differentiation factor 2 (MD-2) complex, where it subsequently delivers intracellular signals [10]. According to the results of some studies, soluble CD14 (sCD14) in the setting of OA and meniscal injury sensitizes FLS to respond to inflammatory stimuli such as TLR ligands. The binding of ligands to TLRs initiates intra-cellular signaling that results in inflammatory gene transcription [11].

During inflammatory conditions, FLSs can be triggered by various extrinsic and intrinsic factors [4]. One of these factors is TLR4, which is one of the major toll-like receptors, expressed in macrophages, monocytes, and granulocytes. However, its additional expression in synoviocytes, osteoblasts, synovial cells, and chondrocytes demonstrates its key role in the pathology of musculoskeletal disorders [12]. It has also been observed that TLR4 is one of the most frequently expressed TLRs in synovial fibroblasts [13]. Furthermore, activation of TLR4 in the synoviocytes of mice induces the influx of pro-inflammatory molecules such as IL­1β and Tumor necrosis factor **(**TNF) [14]. Considering these findings, TLR4 can be contemplated as an essential marker in the pathogenesis of OA, and it may be a possible target for the treatment of OA patients.

One of the most common TLR4 blockers is TAK-242 (Resatorvid), which binds specifically to the intracellular domain of TLR4 to prevent protein-protein association between TLR4 and its adaptor protein. including translocating chain-associated membrane protein (TRAM) and Toll/interleukin-1 receptor domain-containing adaptor protein (TIRAP). Disturbance of the relationship between TLR4 and TIRAP by this cyclohexene derivative (TAK-242) can impair the activation of NF-κB, block subsequent signal transduction, and reduce the levels of inflammatory mediators. Also, TAK-242 binds selectively to TLR4 and interferes with the interaction between TLR4 and TRAM, which can lead to the inhibition of the interferon-sensitive response element (ISRE) and NF-κB activation and downregulation of interferons and cytokines [15]. As is well known, the pathway of NF-κB signaling influences inflammatory reactions by regulating the expression of chemokines, cytokines, and adhesion molecules. As a result, NF-κB pathway dysregulation may have a role in the etiology of inflammatory conditions like OA.

Considering the TAK-242’s suppression effect on TLR4 signaling and subsequently the expression profile of the inflammatory cytokines, it can be considered a potential therapeutic strategy for inflammatory and autoimmune diseases such as OA. Thus, the current report, investigates the efficacy of TAK-242, as a potential anti-inflammatory option, on the mRNA expression levels of IL1-β, IL-6, TNF-α, and TLR4 and also examines its potency on the NF-κB signaling pathway, in FLSs of OA patients.

## Materials and methods

### Subjects

Synovium specimens were obtained from knee OA patients (4 men and 6 women) who were undergoing joint replacement or synovectomy. All patients studied were Iranian, and the mean age of OA patients was 57.44 ± 11.45 years. Tissue samples were collected from Shariati and Laleh hospitals, Tehran, Iran. The patients were end-stage, and the diagnosis was performed based on the 1986 criteria of the American College of Rheumatology (ACR) [16, 17]. An informed consent form to participate in this research was signed by all subjects. The study protocol was reviewed and approved by the ethics committee of Iran University of Medical Sciences (IR.IUMS.FMD.REC.1398.123) and Tehran University of Medical Sciences (IR.TUMS.VCR.REC.1397.037).

### Cell isolation and culture

The knee synovial tissue samples of OA patients were collected from the surgery department in a transporter media containing 15 ml Dulbecco’s modified Eagle’s medium (DMEM, Gibco, Life Technologies, USA) culture medium with 1% penicillin-streptomycin and transferred to the cell culture lab. The tissues were soaked in sterile phosphate-buffered saline (PBS, Gibco Invitrogen, USA) pH (7.3–7.4), alcohol (ethanol 70%), and finally with PBS containing 1% penicillin-streptomycin.The collected tissues were minced into 1 mm X 1 mm pieces in a sterile microplate containing DMEM. The collagenase VIII (1 mg/mL, Sigma–Aldrich, USA) was added to a 50 ml conical centrifuge tube (SPL, Life Sciences, Korea), which contained the dissected synovial tissue fragments, and incubation was performed in a shaker incubator for 80 min 37 °C to promote isolation of FLSs. The samples were vortexed and resuspended two or three times during the incubation.

Then the digested tissues were centrifuged for 10 min at 1000 g, the supernatant was discarded, and the cell pellets were resuspended in 1 mL of complete DMEM before transferring them into two T25 flasks (SPL, Life Sciences, Korea) containing 4 mL of complete DMEM media. The T25 flasks were moved to an incubator with 5% CO2 at 37 °C in a humidified atmosphere. A homogeneous population of FLSs was obtained after three passages. Consequently, for later assessments and interventions, FLS cell passages three through six were employed.

### Identification of FLSs by immunofluorescence staining and flow cytometry

To confirm the isolated cells’ fibroblastic origin, immunofluorescence staining was used. Initially, FLS cells were seeded into a 24-well culture plate and incubated for twenty-four hours at 37 °C. After incubation time the cells were washed with PBS. Next, the cultured FLSs were incubated in cold methanol to fix the cells. Subsequently, the cells underwent a PBS wash and were incubated for one hour on a shaker with a blocking buffer (PBS with Triton-X100 contains 1% BSA). Then, incubation of the cells was performed with the primary antibody, i.e., anti-fibroblast surface protein antibody (ab11333, Abcam, UK), overnight at 4 °C. The secondary antibody, i.e., sheep anti-mouse Ig (human Ig absorb)-FITC conjugated (Ibn Sina, ARI2011F, Iran), was then used to incubate for 60 min at room temperature in a dark environment. To counterstain the nuclei, the 4′, 6-diamidino-2-phenylindole (DAPI) was implemented. Lastly, an inverted fluorescence microscope was implemented for the morphological assessment of stained FLSs.

Several of the primary cell surface CD markers, such as CD13, CD44, CD68, and CD90 [18, 19] were investigated by the flow cytometry technique to further validate the fibroblasts as FLSs. To achieve this goal, FLSs were gathered and subjected to three PBS washings. After that, incubation of the FLSs was performed with the fluorescein isothiocyanate (FITC)-conjugated antibodies against the mentioned surface CD markers at 37 °C for one hour. The antibodies used included anti-CD13 antibody (ab227663), anti-CD44 antibody (ab6124), anti-CD68 antibody (ab31630), and anti-CD90 antibody (ab225), and all of them were purchased from Abcam Inc. (Cambridge, UK). Other FACS analyses used unlabeled cells as the negative control.

### FLS cell grouping and intervention

Every FLS sample was separated into the following 3 study groups: (i) the Untreated group (no treatment), (ii) the LPS group (100 ng/mL), and (iii) the treatment group (TAK-242 (32 µM) + LPS (100 ng/mL)). For determining the effects of TAK-242 on FLSs, pre-treatment of OA-FLSs with TAK-242 (32 µM) was performed in culture media for one hour, and then, the cells were stimulated by LPS (100 ng/mL) for six hours.

### RNA extraction and cDNA synthesis

An RNA extraction kit (SinaColon Co. Tehran, Iran) was used to extract total RNA from each of the three groups according to the manufacturer’s manuals. Then, the purity and yield of extracted RNA were investigated via a NanoDrop spectrophotometer (NanoDrop ND-2000 C Spectrophotometer, Thermo Fisher Scientific, USA) at 260/280 nm absorbance. The extracted RNAs were kept at −80 °C for the next molecular assessment. The first-strand complementary DNA (cDNA) synthesis process was applied to the total RNA using an RNA reverse transcription kit (RT-ROSET, ROJE, Iran).

### Quantitative real-time PCR

The quantitative polymerase chain reaction (qPCR) was adopted using the SYBR Green gene expression master mix and Applied Biosystems StepOnePlus Real-Time PCR System (Foster City, CA, USA). The comparative C_t_ (2-^ΔΔCt^) method was used to calculate the relative mRNA expression levels of the target genes to Glyceraldehyde-3- phosphate dehydrogenase (GAPDH). The primers and the related sequences are provided in Table 1.

**Table 1:** Primer sequences

	Forward	Reverse
IL-1B	ATGGCTTATTACAGTGGCAATGAG	GTAGTGGTGGTCGGAGATTCG
IL-6	AATCATCACTGGTCTTTTGGAG	GGTTATTGCATCTAGATTCTTTGC
TNF	CCTGCCCCAATCCCTTTATT	CCCTAAGCCCCCAATTCTCT
TLR4	AGACCTGTCCCTGAACCCTAT	CGATGGACTTCTAAACCAGCCA
GAPDH	GAGTCAACGGATTTGGTCGT	GACAAGCTTCCCGTTCTCAG

### Protein extraction and western blot

The IκBα and pIκBα protein levels were measured using Western blot analysis. Following a six-hour treatment of the cells with TAK-242 and LPS, the samples were rinsed with ice-cold PBS before being subjected to total protein extraction using the radioimmunoprecipitation assay (RIPA) lysis buffer. This buffer contained whole EDTA-free protease inhibitor cocktail tablets (Roche, Germany). To determine the protein concentrations, the Lowry method was employed. Following that, they were separated using an equivalent volume of protein extracts (50 µg) and sodium dodecyl sulfate-polyacrylamide gel electrophoresis (SDS-PAGE) at 90 V for 2.5 h. Subsequently, the separated proteins were transferred to a PVDF membrane (Thermo Scientific, USA) at 100 V for 90 min. The membrane was incubated with 5% skim milk (Sigma-Aldrich, USA) in 1 X Tris-buffered saline with Tween (TBST) for 60 min at room temperature, to block the free spaces. After that, primary antibodies (anti-IκBα (ab97783, 1:1000), anti-pIκBα (2859, 1:1000), and anti-β-actin (ab8226, 1:1000), Abcam, UK), were used to incubate the membrane overnight at 4 °C. Following the incubation, the membrane was washed with TBST, to eliminate the free antibodies. Horseradish peroxidase-conjugated secondary antibody (PZ5610, 1:3000) was then added, and the blots were incubated for two hours at room temperature. Following the washing of the blots, the protein bands were seen using an enhanced chemiluminescence detection reagent (ECL, GE Healthcare, USA). β-actin was used as an internal control to normalize the results, and the data were semi-quantified using Image J software (NIH, USA).

### Statistical analysis

SPSS software version V26.0 was utilized for analyzing the findings. Also, the GraphPad Prism software V8.0 was used for designing the graphs. Initially, the normality of the data was investigated by the normality test, and it was observed that the data were not normally distributed (Kolmogorov-Smirnov < 0.05). To compare several paired groups, the Friedman test was employed. In order to compare two paired groups, the Wilcoxon signed-rank test was employed. The quantitative data are presented as mean ± standard error of the mean (SEM), and *P*-values less than 0.05 were set to be statistically significant.

## Results

### Identification of FLSs

**Fig. 1:**
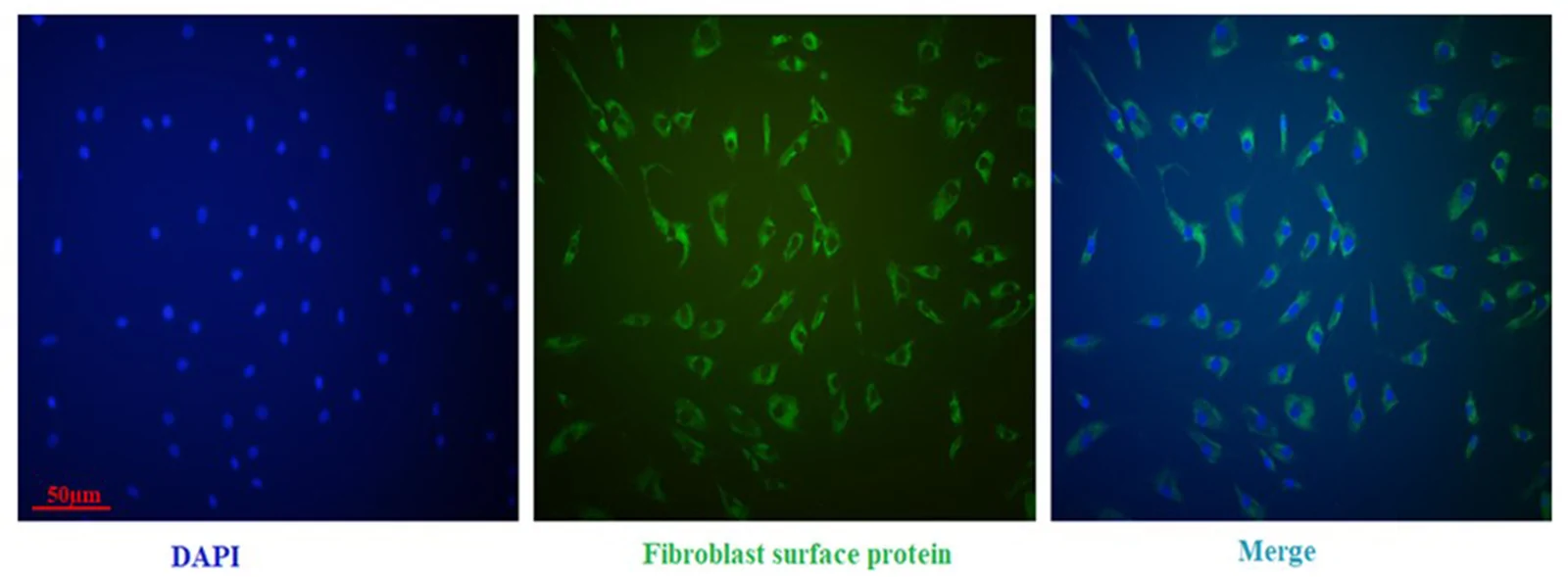
Fibroblast-like synoviocytes (FLSs) were identified using immunofluorescence staining based on fibroblast surface marker expression. The anti-fibroblast surface protein antibody was utilized as the primary antibody, while sheep anti-mouse Ig-FITC conjugated served as the secondary antibody for detection (green color). Additionally, 4′,6-diamidino-2-phenylindole (DAPI) was employed to counterstain nuclei, resulting in a blue coloration

**Fig. 2:**
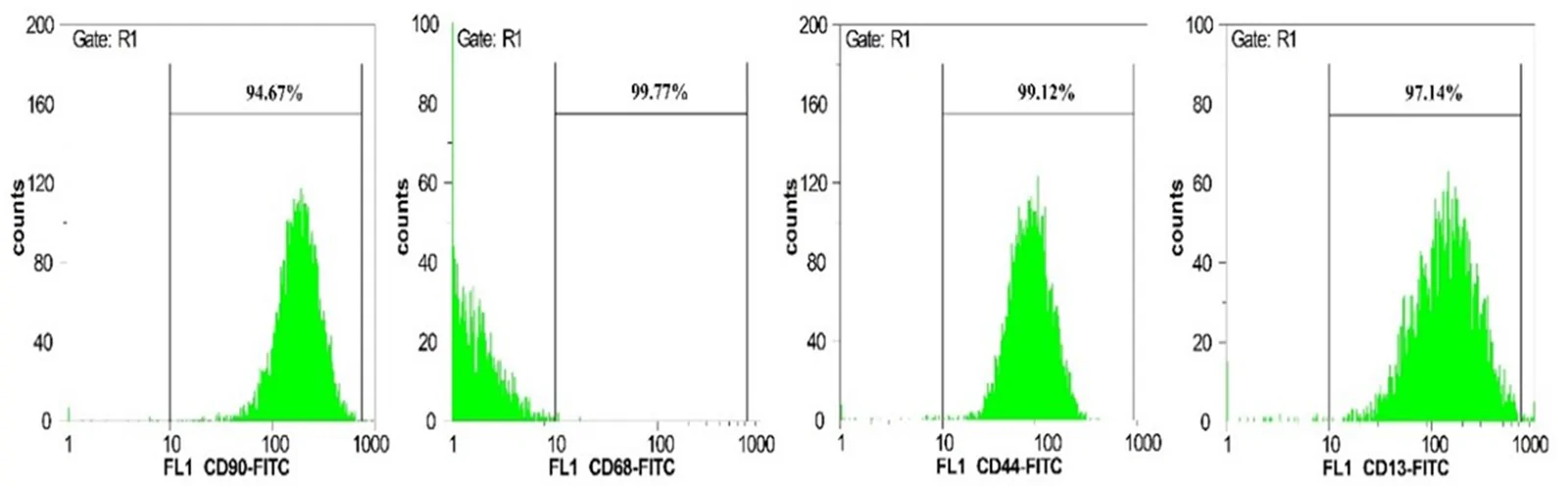
Flow cytometry was employed to detect surface markers (CD13, CD44, CD68, and CD90) of fibroblast-like synoviocytes (FLSs). The results of flow cytometric analysis revealed positive staining for CD13, CD44, and CD90, whereas CD68 displayed negative staining. The purity of the cells was checked after the third passage. This outcome suggests the effective isolation of FLS from synovial tissues

To confirm that isolated cells are FLS, a microscopic evaluation was utilized. After the third passage, immunofluorescence staining was performed. The results of immunofluorescence staining depicted a homogeneous population of fibroblasts (Fig. 1). In the next step, flow cytometry was conducted, the results of which revealed the high expression of cluster of differentiation (CD) 13 (97.14% ± 2.06%), CD44 (99.12% ± 2.21%), and CD90 (94.67% ± 3.7%), and low expression of CD68 (0.23% ± 4.01%) on the surface of isolated cells (Fig. 2). The fact that CD13, CD44, and CD90 were stained positive and CD68 was stained negative, demonstrated the successful separation of FLSs from synovial tissues. Our investigations established that the isolated cells were FLS, and in order to carry out further research, the third and sixth passages were carried out.

### TAK-242 suppresses the expression of pro-inflammatory cytokines and TLR-4 in OA-FLSs

**Fig. 3:**
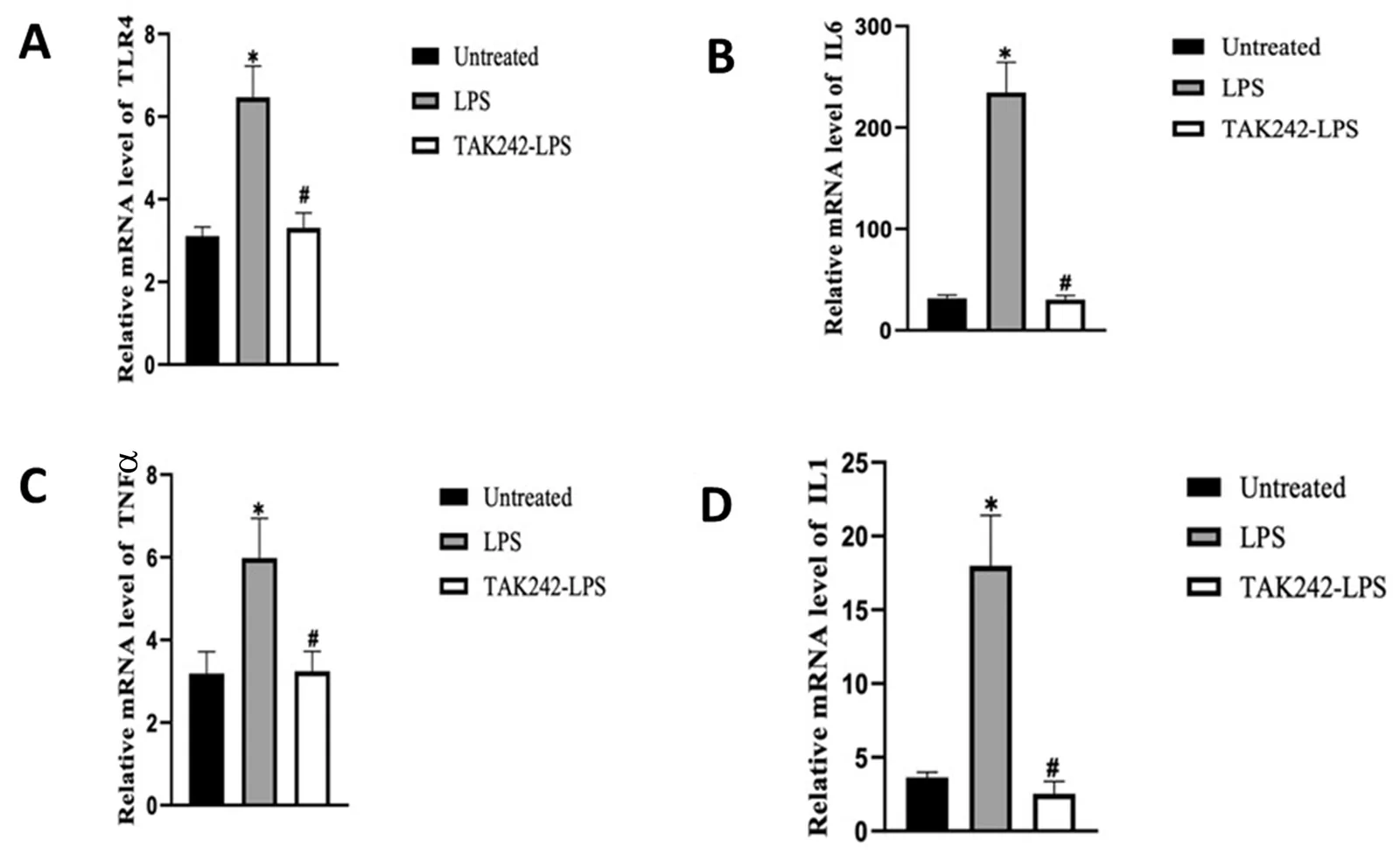
Treating OA FLSs with LPS resulted in a notable increase in the mRNA levels of IL1-β, IL-6, TNF-α, and TLR4 (with p-values of 0.004, 0.016, 0.039, and 0.016, respectively). Conversely, when OA FLSs were treated with TAK-242, there was a significant reduction in the expression of IL1-β, IL-6, TNF-α, and TLR4 (with *p*-values of 0.004, 0.016, 0.039, and 0.004, respectively) compared to the LPS-stimulated groups. *Abbreviations OA* Osteoarthritis, *IL* Interleukin, *TLR* Toll Like Receptor, *FLS* Fibroblast Like synoviocytes, *TNF* Tumor necrosis factor, *LPS* lipopolysaccharides

The baseline expressions (Average ± SD) of the *IL1-β* (3.51 ± 1.15), *IL-6* (31.73 ± 8.97), *TNF-α* (2.91 ± 1.39), and *TLR4* (3.11 ± 0.62) genes were compared with the expression levels of these genes after treatment of LPS-stimulated FLSs (Fig. 3). Treating FLSs with LPS led to a significant increase in the mRNA levels of *IL1-β* (17.99 ± 10.21, *p* = 0.004), *IL-6* (234.53 ± 84.57, *p* = 0.016), *TNF-α* (5.98 ± 2.72, *p* = 0.039), and *TLR4* (6.47 ± 2.36, *p* = 0.016) in OA FLSs. On the other hand, treatment with TAK-242 led to a significant reduction in the expression pattern of *IL1-β* (2.54 ± 2.62, *p* = 0.004), *IL-6* (30.49 ± 11.90, *p* = 0.016), *TNF-α* (3.24 ± 1.36, *p* = 0.039), and *TLR4* (3.31 ± 1.07, *p* = 0.004) in OA FLSs group in comparison with the LPS-stimulated groups. Collectively, the results showed that TAK-242 may be able to suppress inflammatory responses through downregulation of proinflammatory cytokine production in LPS-stimulated FLSs.

### TAK-242 represses TLR4/NF-κB signaling pathway in OA-FLSs

**Fig. 4:**
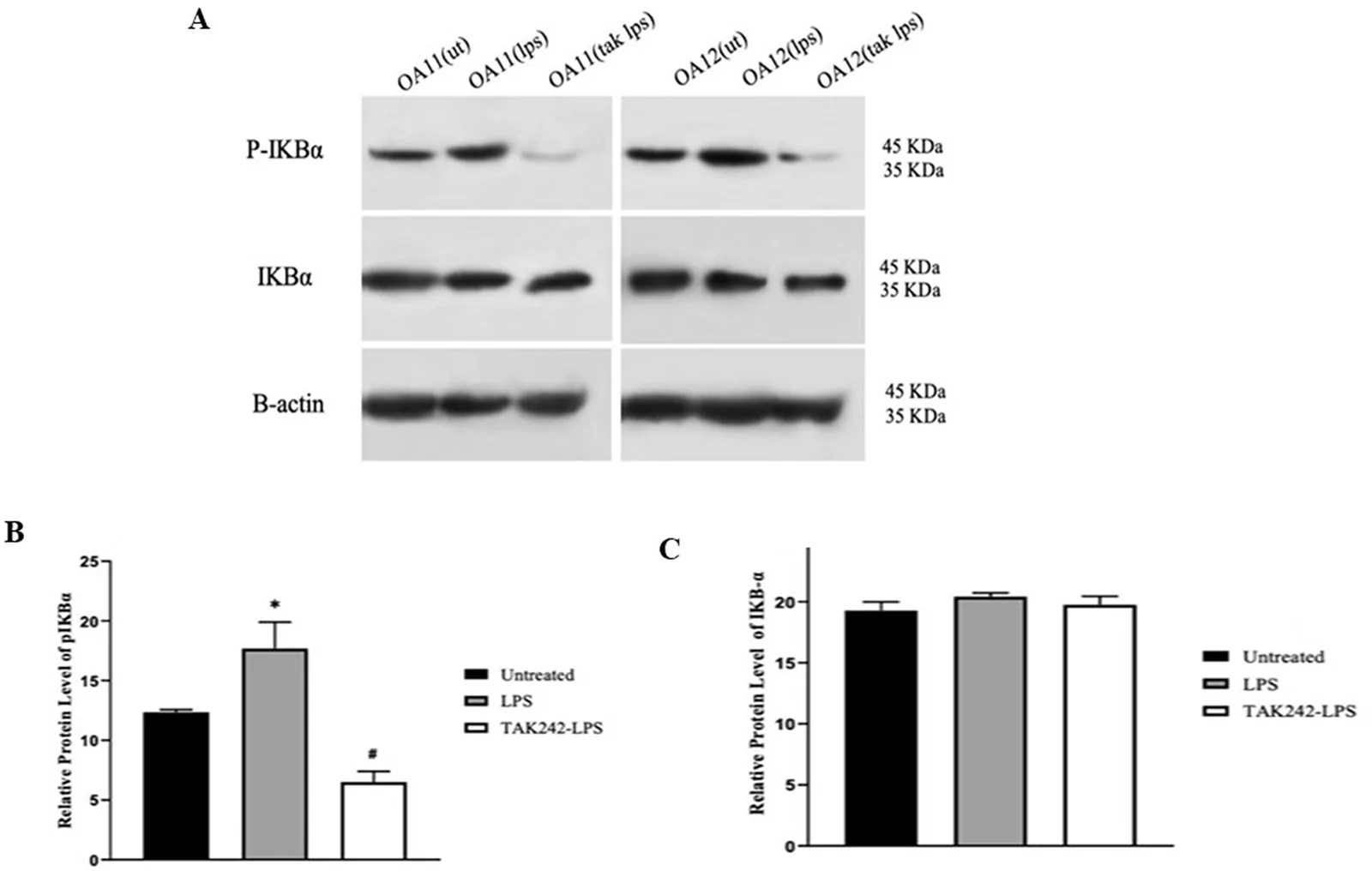
**A** The westernblot on P-IKB-α and IKBα proteins. **B** LPS-stimulated OA-FLSs indicated a significant increase in pIκBα in comparison with the untreated group, and pretreatment with TAK-242 led to a significant decrease in pIκBα in OA-FLSs in comparison with the LPS group (**A**, **B**). **C** LPS and TAK-242 treatment did not affect IκBα protein expression (**A**, **C**)

For investigating the molecular mechanisms associated with the anti-inflammatory effects of TAK-242, an assessment of major proteins with vital roles in the TLR4/NF-κB signaling pathway was also performed. As a result, FLSs were pretreated with TAK-242, and LPS was then used to stimulate the cells. To assess the protein levels of IκBα and pIκBα, a western blot was then carried out (Fig. 4). LPS-stimulated OA-FLSs indicated a significant increase in pIκBα in comparison with the untreated group. On the other hand, a significant decline in pIκBα in OA-FLSs in comparison with the LPS group, was observed during pretreatment with TAK-242. Thus, TAK-242 could inhibit the LPS-induced phosphorylation of IκBα. According to these results, TAK-242 may reduce the expression of pro-inflammatory mediators in LPS-stimulated FLSs through inhibition of FLSs’ TLR4/NF-κB pathway.

## Discussion

Osteoarthritis, also known as OA, is a persistent degenerative condition affecting the joints, distinguished by the deterioration of cartilage, the presence of inflammation, as well as the occurrence of pain and stiffness. FLS stem from the mesenchymal lineage, and following joint damage, synovial cells respond to a range of inflammatory cytokines and matrix degradation products by amplifying NF-κB-dependent signaling pathways, producing more inflammatory mediators that eventually lead to cartilage destruction and bone erosion [20]. It has been demonstrated that the TLR4/NF-κB signaling pathway has a major involvement in the development of chronic inflammation that is present in OA patients [21]. Besides, it has been reported that TLR4 is overexpressed on OA-FLSs [22].

Our results showed that TAK-242 reduces the expression levels of TLR4 in LPS-stimulated FLSs which is consistent with other studies [15].

IL-1 is a cytokine that has long been considered crucial in the pathogenesis of OA considering its roles in degrading cartilage, driving inflammation, and even upregulating nerve growth factors. IL-6 is another pro-inflammatory cytokine that is indicated to be increased in the synovial fluid of patients with OA [23]. The synthesis and secretion of this cytokine, along with other pro-inflammatory factors such as TNF-α, is supported by the IL-1β-activated NF-κB pathway [24].

IL-1β exerts a stimulatory effect on the secretion of IL-6 and other pro-inflammatory cytokines, which potentiate the catabolic effects of IL-1β and, at the same time, serve as catabolic mediators on their own [24]. IL-6 attracts inflammatory cells and causes synovial inflammation, respectively, resulting in the even greater production and secretion of IL-1β. Furthermore, TNF-α increases the synthesis of IL-6, and IL-8, regulated on activation of normal T cell expressed and secreted (RANTES), and vascular endothelial growth factor (VEGF), and together with the already mentioned IL-1β, this factor induces the production of inducible nitric oxide synthase (iNOS), cyclooxygenase 2 (COX-2), and prostaglandin E2 (PGE-2) synthase, which further upregulates IL-1β and TNF-α production [5].

IL-6 is regarded to be more important in the pathology of OA due to its ability to stimulate the production of matrix metalloproteinases (MMPs), which are enzymes that degrade cartilage. In addition to its effects on cartilage, IL-6 promotes synovitis, or inflammation of the synovial membrane. Furthermore, IL-6 regulates bone remodeling, which is disrupted in OA. IL-6 stimulates osteoclast formation and activity, leading to bone resorption and contributing to the development of osteophytes, or bony outgrowths, which are a hallmark of OA.

TNF-α promotes the generation of other pro-inflammatory cytokines, such as IL-1β and IL-6, which can induce the production of matrix metalloproteinases (MMPs) and aggrecanases, enzymes that degrade cartilage. Furthermore, TNF-α regulates bone remodeling, which is disrupted in OA. It stimulates the differentiation of osteoclasts, cells that resorb bone, leading to bone loss and the development of osteophytes, or bony outgrowths, a hallmark of OA. TNF-α also inhibits the differentiation and activity of osteoblasts, cells that form new bone [25].

Our results have revealed that TAK-242 can inhibit the expression of IL-1β, IL-6, and TNF-α significantly. In a similar study, TAK-242 showed a trend of inhibiting the increased expression of IL-6, IL-8, MMP-1, and VEGF in LPS-stimulated MH7A cells in a dose-dependent manner, which is in line with our findings [26].

The possible molecular mechanism for the efficacy of TAK-242 might be through the inhibition of the NF-κB pathway which is downstream of TLR4 [27] and activated abnormally in OA patients. NF-κB is a central pathway in pro-inflammatory cytokine production [28]. NF-κB coordinates aberrant cartilage catabolic pathways by inducing the expression of matrix-degrading enzymes and other OA-associated factors. This process is promoted by the overexpression of various catabolic genes that have NF-κB-dependent response elements in their promoters like MMP1, MMP9, and ADAM metallopeptidase with thrombospondin type 1 motif 5 (ADAMTS5) [29–34], as well as the enhancement of the expression of major pro-inflammatory and destructive mediators of OA, including COX2, PGE2, and iNOS [35–40].

Our findings demonstrated that TAK-242 reduces the phosphorylation level of IκBα and inhibits the NF-κB signaling, which can lead to the downregulation of pro-inflammatory gene expression in the FLSs. Our findings are in line with the study by Samarpita et al. [41].

Soluble biglycan (sBGN), a potential mediator of cartilage degradation in osteoarthritis, is an essential component of extracellular matrix which has been shown to possess proinflammatory properties. Blocking TLR4 by using a low molecular weight inhibitor of TLR4 signaling (CLI095, also known as TAK-242) abrogated the sBGN-induced increase of *TLR4* mRNA expression [42].

Results of another study demonstrated that TAK242 was the only inhibitor able to block significantly serum amyloid A (A-SAA)-inducing cytokines and MMPs expression in chondrocytes and FLS. It strongly suggests that A-SAA is an endogenous TLR4 agonist in OA. They concluded that systemic or local A-SAA expression inside OA joint cavity may play a key role in inflammatory process seen in osteoarthritis, which could be counteracted by TLR4 inhibition [43]. Results of a study by Lambert et al. showed that Coll2-1 peptide (a type II collagen peptide) significantly increased IL-8 gene expression and production by synoviocytes, and CLI-095 significantly decreased IL-8 expression. Coll2-1 activates synoviocytes to produce IL-8 and induces arthritis in rat. These findings suggest that neutralizing Coll2-1 could be a therapeutic approach of arthritis [44].

## Conclusion

In conclusion, the current study supports that TAK-242, as a specific antagonist of TLR4, effectively modulates the TLR4-mediated inflammatory responses in OA-FLSs through the inhibition of the NF-κB signaling pathway and can be considered a possible therapeutic target for OA patients. Considering the lack of response to conventional and biological treatments in some OA patients, TLR4 inhibitors, especially TAK-242, could be a therapeutic candidate for controlling inflammatory responses and related complications.

### Study limitation

We have some limitations such as lack of access to synovial tissues of patients and also lack of access to synovial tissues of OA patients with different stages to compare gene expression between stages.

## Data Availability

All data generated or analyzed during this study are available upon request. .
